# Correlation analysis between plasma concentration of nilotinib and clinical efficacy and safety in patients with chronic myeloid leukemia: a single–center retrospective cohort study

**DOI:** 10.3389/fphar.2025.1676800

**Published:** 2025-09-05

**Authors:** Ying Liu, Taoyan Lin, Boxin Zhao, Yilei Li, Ping Zheng

**Affiliations:** 1 Department Pharmacy, Institute Nanfang Hospital, Organization Southern Medical University, Guangzhou, China; 2 Department Clinical Pharmacy Center, Institute Nanfang Hospital, Organization Southern Medical University, Guangzhou, China

**Keywords:** nilotinib, chronic myeloid leukemia, plasma concentration, TDM, efficacy, safety

## Abstract

**Objective:**

This study aimed to investigate the correlation between nilotinib plasma concentrations and clinical efficacy and safety in patients with chronic myeloid leukemia (CML), thereby supporting personalized therapeutic optimization.

**Methods:**

We conducted a retrospective cohort study of 121 CML patients receiving nilotinib with therapeutic drug monitoring (TDM) at Nanfang Hospital of Southern Medical University between March 2021 and February 2024. Major molecular response (MMR) and adverse events (CTCAE v5.0) were analyzed against concentrations. Receiver operating characteristic (ROC) curves defined thresholds; dose-normalized exposure (C_norm_ = C_min_/(Dose/600 mg)) addressed TDM bias.

**Results:**

The effective group showed higher nilotinib concentrations than those in the ineffective group (1,036.40 ± 463.67 vs. 737.14 ± 518.97 ng mL^-1^; P < 0.001), confirmed post-normalization (1,045.10 ± 468.08 vs. 858.34 ± 723.66 ng mL^-1^; P < 0.05). The ROC-derived efficacy threshold was 636.99 ng mL^-1^ (AUC = 0.693, 95% CI: 0.596–0.791), which was identical after normalization (AUC = 0.655, 95% CI: 0.554–0.755). Although adverse events were common (76.9% of patients), they showed no overall concentration dependence (P = 0.288). However, hyperbilirubinemia risk was significantly elevated in patients with concentrations >1,273.98 ng mL^-1^ cohort (50.0% vs. 20.0%–22.6%; P = 0.030), with a toxicity threshold identified at 1,290.34 ng mL^-1^ (AUC = 0.656, 95% CI: 0.540–0.771). Longer treatment duration was also associated with higher drug exposure (P = 0.029).

**Conclusion:**

Nilotinib concentrations predict MMR attainment independent of TDM-driven dose adjustments (validated via C_norm_). We recommend targeting early-phase concentrations >636.99 ng mL^-1^, monitoring bilirubin above 1,290.34 ng mL^-1^, and integrating dynamic TDM with pharmacogenetic profiling.

## 1 Introduction

Chronic myeloid leukemia (CML) is a malignant neoplasm characterized by the clonal proliferation of hematopoietic stem cells driven by the breakpoint cluster region–Abelson (BCR::ABL1) fusion gene (Alves et al., 2021; Deininger et al., 2020; Garcia-Ferrer et al., 2019), with more than 90% of patients exhibiting Philadelphia chromosome (Ph) abnormalities (Tian et al., 2018). Although tyrosine kinase inhibitors (TKIs) have substantially improved clinical outcomes, clinical evidence indicates that some patients develop resistance, experience intolerance, or show no response to first-generation BCR::ABL1 inhibitors such as imatinib (Sampaio et al., 2021; García-Gutiérrez and Hernández-Boluda, 2019; Dinić et al., 2024; He et al., 2021).

To optimize therapeutic strategies and improve remission rates in CML, several second-generation BCR::ABL1tyrosine kinase inhibitors (TKIs), including nilotinib, dasatinib, ponatinib, and bosutinib, have been successfully developed (Sampaio et al., 2021; Tian et al., 2018). More recently, asciminib, a groundbreaking treatment for CML, has gained FDA approval as the first STAMP inhibitor, which offers a novel mechanism of action by targeting the myristoyl pocket of BCR::ABL1, proves effective in overcoming resistance to traditional TKIs, and demonstrates superior efficacy and a favorable safety profile in clinical trials (Akhtar et al., 2025; Choi, 2023). Notably, nilotinib exhibits approximately 20-fold greater inhibitory potency compared to imatinib, with around 50% of imatinib-resistant patients achieving complete cytogenetic response (CCyR) while maintaining acceptable tolerability (Dinić et al., 2024; He et al., 2021; Wang et al., 2018).

Nilotinib, a second-generation BCR::ABL1 inhibitor, is primarily indicated for patients with chronic-phase or accelerated-phase CML who exhibit resistance or intolerance. It shows a bioavailability of 30%, with 98% plasma protein binding and predominant hepatic metabolism via CYP3A4 (Ding and Zhong, 2013; Tian et al., 2018). According to the 2024 National Comprehensive Cancer Network (NCCN) Clinical Practice Guidelines in Oncology for Chronic Myeloid Leukemia (Shah et al., 2024), nilotinib is recommended as first-line therapy for patients with intermediate-to high-risk CML (Fukuda, 2022; Wang et al., 2018). However, considerable interindividual variability in plasma concentration has been reported worldwide, and a consensus therapeutic window for nilotinib has not yet been established. Therapeutic drug monitoring (TDM), a clinical approach for quantifying drug concentrations in blood and evaluating therapeutic ranges, is recognized as a key strategy for optimizing nilotinib therapy (Miura, 2015; Mueller-Schoell et al., 2021; Tuzimski and Petruczynik, 2020). Therefore, investigating the relationship between nilotinib plasma concentrations and clinical efficacy and safety profiles in CML patients provides essential evidence for assessing the feasibility of TDM implementation.

This study investigates the clinical significance of nilotinib plasma concentrations in CML patients by evaluating efficacy and safety outcomes. Through detailed analysis of the complex relationships between plasma concentrations, therapeutic responses, and adverse drug reactions (ADRs), and by systematically examining the influence of factors such as gender, age, treatment duration, and hepatic or renal function indices, this study aims to provide essential evidence to support personalized nilotinib treatment strategies in CML management.

## 2 Materials and methods

### 2.1 Study design and participants

This retrospective cohort study was conducted at Nanfang Hospital of Southern Medical University between March 2021 to February 2024. The study included CML patients receiving oral nilotinib therapy with TDM. Inclusion criteria were: (1) a confirmed diagnosis of CML; and (2) ongoing nilotinib treatment with plasma concentration monitoring. A total of 123 CML patients treated with nilotinib were initially considered.

**FIGURE 1:**
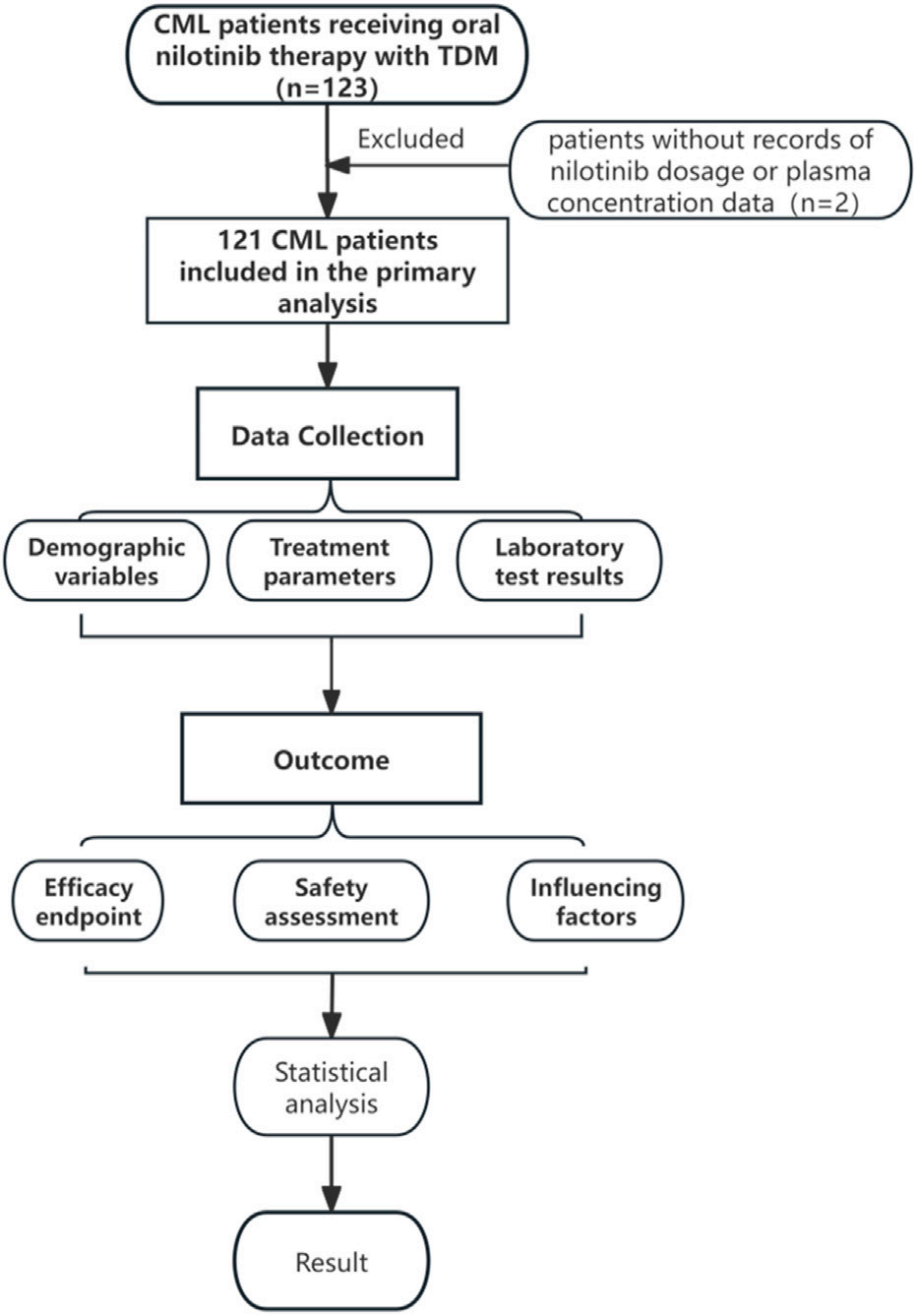
Flowchart of the study design.

Exclusion criteria included patients with missing records of nilotinib dosage or plasma concentration data. Subsequently, patients lacking crucial data (n = 2) were excluded, yielding 121 patients included in the primary analysis. The study protocol was approved by the Institutional Review Board (Approval No: NFEC–2025–147). The study flowchart is presented in Figure 1.

### 2.2 Analytical assays

An ultra-performance liquid chromatography-tandem mass spectrometry (UPLC-MS/MS) method was employed to quantify nilotinib plasma concentrations. The UPLC-MS/MS system was calibrated using a series of standard solutions, and a calibration curve was generated with a linear regression analysis (y = mx + b, R^2^ > 0.99), where y represents the peak area ratio of the analyte to the internal standard, and x represents the concentration of the analyte. Quality control samples were analyzed to verify the accuracy and precision of the assay:Lower limit of quantification (LLOQ): 1 ng mL^-1^;Linear range: 1–5,000 ng mL^-1^;Intra-/inter-day precision: <15% CV. Concentration normalization was performed using an internal standard to account for potential matrix effects and instrument variability.

Sampling Protocol: Blood samples were strictly collected within 30 min prior to the next scheduled dose (trough concentration, C_min_) between 8:00–8:30 a.m., ensuring measurement of true steady-state concentrations after ≥8 days of continuous dosing. This sampling time was selected to target the trough concentration based on the known pharmacokinetic profile of nilotinib, which reaches peak serum concentration (C_max_) approximately 4 h post-dose, has an elimination half-life (t_1/2_) of approximately 16 h, and achieves steady-state plasma concentrations within 8 days of continuous administration (Ding and Zhong, 2013; Wang et al., 2018).

Analysis of Repeated Measures: Pharmacokinetic data were analyzed using a mixed-effects model to account for both inter-individual and intra-individual variability. The model included time as a fixed effect and individual random intercepts and slopes to capture the variability in concentration profiles among participants. Model fit was evaluated using the Akaike Information Criterion (AIC) and Bayesian Information Criterion (BIC) (Jones, 2011), with lower values indicating a better fit. Nonlinear mixed-effects modeling was conducted to estimate the structural model and population pharmacokinetic parameters and pharmacokinetic parameter estimates (Choi et al., 2020; Kang and Lee, 2009).

### 2.3 Efficacy and safety evaluation

According to the NCCN 2024 guidelines (Shah et al., 2024), major molecular response (MMR) was used to evaluate therapeutic efficacy, defined as BCR::ABL1 transcript levels ≤0.1% on the International Scale (BCR::ABL1 IS) at any time point. Adverse events were graded based on the Common Terminology Criteria for Adverse Events (CTCAE) (Freites-Martinez et al., 2021) version 5.0, with systematic monitoring and documentation throughout the treatment period.

### 2.4 Data collection

Patient demographic data, including age, sex, prior treatment, and comorbidities, were collected. Information on nilotinib dosing regimens, plasma concentration measurements, BCR:ABL1 IS levels, alanine aminotransferase (ALT), aspartate aminotransferase (AST), total bilirubin (TBIL), serum creatinine (Cr), neutrophils (NEUT), red blood cell (RBC), hemoglobin (Hb), platelets (PLT), triglycerides (TG), total cholesterol (TC), low-density lipoprotein cholesterol (LDL-C), glucose (GLU), amylase, lipase, as well as therapeutic efficacy outcomes and occurrences of adverse events during treatment, was also recorded.

Data on BCR::ABL1 mutation status at baseline or during treatment were not routinely collected as part of standard clinical care for all patients in this cohort and are therefore unavailable for analysis. Similarly, pharmacogenetic profiling (e.g., for CYP3A4, CYP2C8, or UGT1A1 polymorphisms) and comprehensive screening for drug-drug interactions were not performed. While these factors represent potential confounders, their absence is a inherent constraint of the retrospective design. Future prospective studies are warranted to incorporate these valuable parameters.

### 2.5 Statistical analysis

Statistical analyses were conducted using IBM SPSS Statistics (version 21.0, IBM Corp., Armonk, NY, United States). Continuous variables were described as mean ± standard deviation (Mean ± SD), and categorical variables were presented as percentages. Comparative analyses were performed using the X^2^ test, the Mann-Whitney U test, and the Kruskal-Wallis H test. Spearman’s rank correlation was used to assess associations. Receiver operating characteristic (ROC) curve analysis was applied to evaluate the predictive value of plasma concentrations for therapeutic efficacy and safety. Univariate and multivariate linear regression models were used to identify factors influencing nilotinib plasma concentrations. Data visualization was performed using GraphPad Prism (version 10.0, GraphPad Software, San Diego, CA, United States). A two-tailed p < 0.05 was considered statistically significant.

## 3 Results

### 3.1 Baseline

Patients were stratified into the effective group (n = 67) and the ineffective group (n = 54) based on achievement of MMR. Baseline characteristics of the two groups are presented in Supplementary Table S1. No significant differences were observed between the groups in baseline variables, including sex, age, prior treatment, comorbidities, and dosage (P > 0.05). However, significant differences were noted in treatment duration (P = 0.04).

### 3.2 Relationship between plasma concentration and clinical efficacy

**FIGURE 2:**
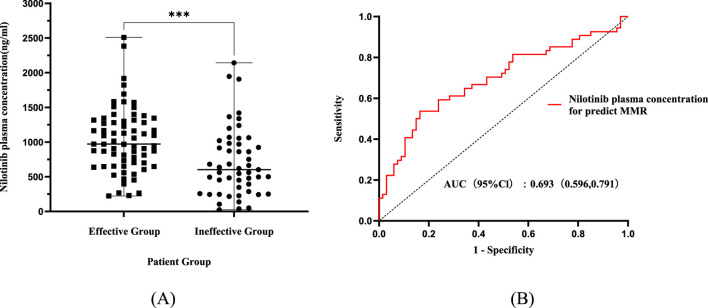
Assessment of nilotinib plasma concentration and its predictive value for treatment efficacy. **(A)** Trough plasma concentrations were significantly higher in patients who achieved major molecular response (Effective group) compared to those who did not (Ineffective group). Data are presented as mean ± SD. ***P < 0.001. **(B)** Receiver operating characteristic (ROC) curve analysis of nilotinib plasma concentration for predicting major molecular response (MMR). The area under the curve (AUC), optimal efficacy threshold (636.99 ng/mL), and its corresponding sensitivity and specificity are indicated. Abbreviations: AUC, area under the curve; CI, confidence interval.

The median plasma concentration of nilotinib among the 121 patients was 870.04 ng mL^-1^ (IQR:526.35–1,219.97), with a mean value of 902.84 ± 509.42 ng mL^-1^. The nilotinib plasma concentrations were significantly higher in the effective group (1,036.40 ± 463.67 ng mL^-1^) compared to the ineffective group (737.14 ± 518.97 ng mL^-1^) (P < 0.001; Figure 2A). A significant moderate positive correlation was observed between nilotinib plasma concentrations and MMR (Spearman’s ρ = 0.33, P < 0.001).

Using MMR attainment as the dependent variable and plasma concentration as the independent variable, ROC curve analysis yielded an area under the curve (AUC) of 0.693 (95% CI:0.596–0.791) for predicting MMR. At the maximal Youden index (J = 0.373), the optimal plasma concentration threshold was identified as 636.99 ng mL^-1^, with a sensitivity of 83.6% and a specificity of 53.7% (Figure 2B).

#### 3.2.1 Dose normalization to address TDM confounding

To mitigate potential bias from TDM-guided dose adjustments (where low-exposure patients receive higher doses and *vice versa*), we applied dose normalization using the formula:

$$\text{Cnorm}=\text{Cmin}/\left(\text{Dose}/600\text{mg}\right)$$

**FIGURE 3:**
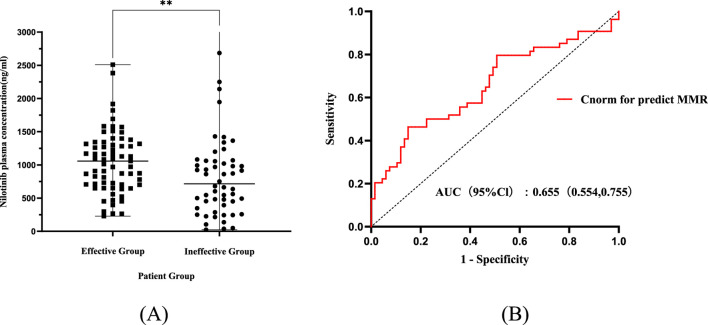
Analysis of dose-normalized nilotinib exposure and its predictive value for treatment efficacy. **(A)** Comparison of dose-normalized plasma concentrations (Cnorm) between the effective and ineffective groups. Cnorm was calculated as Cmin/(Dose/600 mg) to eliminate bias introduced by TDM-guided dose adjustments. Data are presented as mean ± SD. **P < 0.01. **(B)** Receiver operating characteristic (ROC) curve analysis of Cnorm for predicting major molecular response. The persistence of a significant AUC after dose normalization strengthens the finding that the efficacy threshold is a physiologically relevant target, independent of administered dose. Abbreviations: AUC, area under the curve; CI, confidence interval; Cnorm, dose-normalized concentration.

After normalization, median C_norm_ was 907.02 ng mL^-1^ (IQR: 546.14–1,274.47), mean 907.02 ± 600.41 ng mL^-1^. The efficacy correlation persisted:MMR-achievers maintained significantly higher C_norm_ (1,045.10 ± 468.08 ng mL^-1^) versus non-responders (858.34 ± 723.66 ng mL^-1^; P < 0.05, Figure 3A), with a sustained moderate correlation to response depth (ρ = 0.27, P < 0.05).

ROC analysis using C_norm_ showed an AUC of 0.655 (95% CI:0.554–0.755) and notably identified the identical optimal threshold of 636.99 ng mL^-1^ (sensitivity 85.1%, specificity 46.3%; Figure 3B). This consistency confirms that while TDM practices attenuated statistical associations, the underlying exposure-efficacy relationship remains robust.

### 3.3 Relationship between plasma concentration and overall incidence of ADRs

**FIGURE 4:**
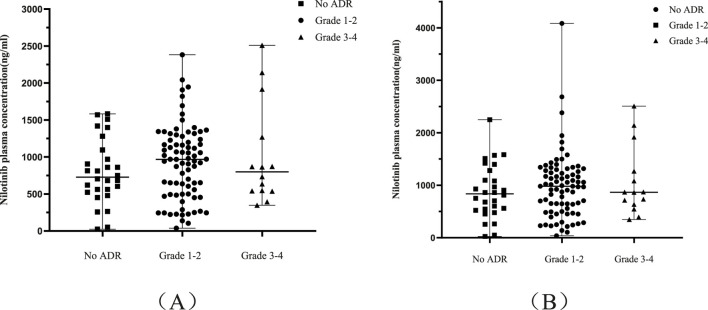
Analysis of the relationship between nilotinib exposure and the incidence and severity of ADRs. **(A)** Comparison of raw trough plasma concentrations (Cmin) among patients with no ADRs, Grade 1–2 ADRs, and Grade 3–4 ADRs. **(B)** Comparison of dose-normalized concentrations (Cnorm) among the same patient groups. Data are presented as mean ± SD. Ordinary one-way ANOVA revealed no statistically significant differences in nilotinib exposure across all comparison groups (all P > 0.05), indicating that plasma concentration was not a predictor of ADR risk or severity. This finding was consistent for both raw **(A)** and dose-normalized **(B)** concentrations, and aligns with the clinical profile of nilotinib where ADRs are common but predominantly low-grade and manageable. Abbreviations: ADR, adverse drug reaction; Cnorm, dose-normalized concentration.

Among 121 CML patients, 93 (76.9%) experienced treatment-emergent adverse events; however, the vast majority (65.29% of all patients) were Grade 1–2 in severity, with only 11.57% experiencing Grade 3–4 events. Initial analysis revealed no significant difference in mean nilotinib concentrations between patients with ADRs (934.90 ± 527.89 ng mL^-1^) and those without (796.39 ± 434.23 ng mL^-1^; P = 0.288). Non-hematologic toxicities predominated, primarily grade 1–2 hyperbilirubinemia (28.1%), ALT elevation (26.4%), and hypercholesterolemia (24.0%), with only 0.8% progressing to grade 3–4 for each. Hematologic toxicities included anemia (25.7%) and thrombocytopenia (13.2%), exhibiting grade 3–4 incidences of 1.7% and 2.5% respectively. Importantly, no concentration-dependent severity gradient was observed: grade 1–2 ADRs (n = 79, 65.3%) occurred at 921.09 ± 498.23 ng mL^-1^ while grade 3–4 events (n = 14, 11.6%) manifested at 1,012.81 ± 689.23 ng mL^-1^ (P > 0.05, Figure 4A).

This pattern persisted after dose normalization, where neither overall ADR incidence (ADR group: 988.66 ± 624.35 ng mL^-1^ vs. not-ADR group: 872.53 ± 513.03 ng mL^-1^; P = 0.721) nor severity stratification (grade 1–2: 975.22 ± 620.03 ng mL^-1^ vs. grade 3–4: 1,064.22 ± 666.92 ng/mL; P > 0.05, Figure 4B) showed no statistically significant associations. Fourteen patients (11.57%) required dose modifications (reduction n = 7, discontinuation n = 7) due to intolerance, though no fatal events occurred. Collectively, these analyses demonstrate that nilotinib exposure levels–whether raw or dose-normalized–were not predictive of ADR risk or severity in this cohort.

Among all ADRs, non-hematologic toxicities were predominant, with the most common being hyperbilirubinemia (28.1%), elevated ALT (26.4%), and hypercholesterolemia (24.0%), primarily grade 1–2 (27.3%, 25.6%, and 24.0%, respectively). Only one case (0.8%) of grade 3–4 toxicity was reported for either hyperbilirubinemia or elevated ALT. Hematologic toxicities mainly included anemia (25.7%) and thrombocytopenia (13.2%), with grade 3–4 incidences of 1.7% and 2.5%, respectively (Supplementary Table S2).

### 3.4 Relationship between the incidence of ADRs in different concentration range groups and the plasma concentration

**FIGURE 5:**
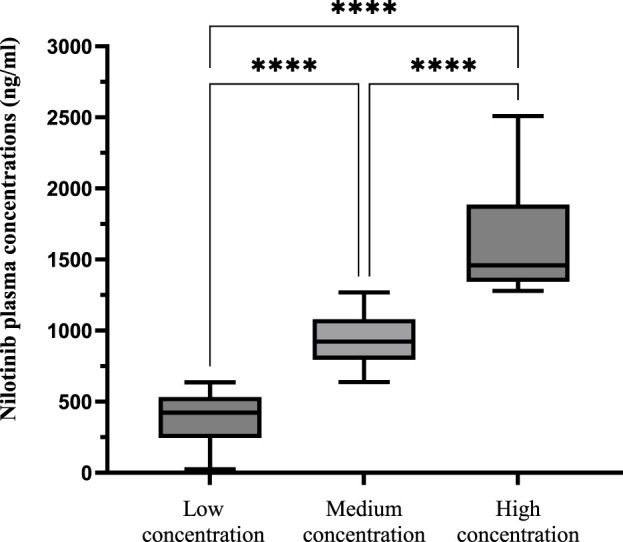
Relationship between Plasma Concentration of ADRs in Different Concentration Ranges. Patients were stratified into three cohorts based on pharmacokinetic-derived thresholds: low (<636.99 ng/mL, n = 40), medium (636.99–1,273.98 ng/mL, n = 53), and high (>1,273.98 ng/mL, n = 28) concentration groups. The overall incidence of ADRs was high and comparable across all groups (72.5%–79.2%), with Grade 1–2 events being predominant in each cohort. The distribution of most specific ADR types (e.g., rash, elevated ALT, anemia) did not differ significantly among groups (all P > 0.05). A notable exception was hyperbilirubinemia, which exhibited a significant concentration-dependent increase in incidence (P = 0.030), with the high-concentration group experiencing a markedly higher rate (50.0%). Abbreviations: ADR, adverse drug reaction.

Based on the efficacy-derived threshold (636.99 ng mL^-1^)^1^ and pharmacokinetic characteristics (Ding and Zhong, 2013; Tian et al., 2018) suggesting toxicity risk escalates above double this threshold (>1,273.98 ng mL^-1^), patients were stratified into three cohorts: low concentration (<636.99 ng mL^-1^, n = 40), medium concentration (636.99–1,273.98 ng mL^-1^, n = 53) and high concentration (>1,273.98 ng mL^-1^, n = 28). The overall incidences of ADRs were 72.5%, 79.2% and 78.6%, respectively. Significant differences in plasma concentration distribution were observed among the three groups (P < 0.0001; Figure 5). In all cohorts, grade 1–2 ADRs were predominant (57.5%, 69.8%, 67.9%), while grade 3–4 events were less frequent (15.0%, 9.4%, 10.7%).

The distributions of ADRs, including rash, headache, myalgia, elevated ALT, elevated AST, increased lipase, increased amylase, elevated triglyceride, elevated cholesterol, thrombocytopenia, anaemia, and neutropenia, showed no significant differences among the groups (all P > 0.05). However, a significant association was observed between nilotinib concentration groups and the incidence of hyperbilirubinemia (P = 0.030). The high-concentration group exhibited a markedly higher incidence (50.0%) and severity of hyperbilirubinemia compared to the low (20.0%) and medium-concentration groups (22.6%), with grade 3–4 events occurring in 3.6% of high-concentration group, while no severe cases were reported in the other groups (Supplementary Table S3).

**FIGURE 6:**
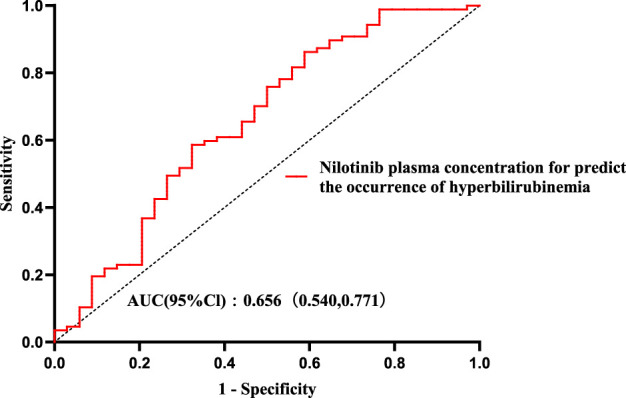
Receiver operating characteristic (ROC) curve analysis for identifying a nilotinib plasma concentration threshold associated with hyperbilirubinemia. The ROC curve was generated to evaluate the predictive performance of nilotinib plasma concentration for the occurrence of hyperbilirubinemia. The area under the curve (AUC) was 0.656 (95% CI: 0.540–0.771). The optimal predictive threshold was identified as 1,290.34 ng/mL. Abbreviations: AUC, area under the curve; CI, confidence interval.

Utilising the occurrence of hyperbilirubinemia as the dependent variable and plasma concentration as the independent variable, ROC curve analysis yielded an AUC of 0.656 (95% CI: 0.540–0.771) for predicting elevated TBIL. At the maximal Youden index (J = 0.274), the optimal plasma concentration threshold was identified as 1,290.34 ng mL^-1^, resulting in a sensitivity of 41.2% and a specificity of 86.2% (Figure 6).

### 3.5 Treatment duration and clinical response

We compared the treatment duration between the effective group and the ineffective group. The distribution of patients across different treatment phases (0–6 months, 6–12 months, >12 months) was significantly different between the two cohorts (P < 0.001). A notably larger proportion (67.2%) of the effective group were found in the longer treatment duration groups (>12 months), whereas the ineffective group were more prevalent in the shorter treatment duration groups (0–6 months, 44.0%). This indicates that responders were consistently exposed to nilotinib for a longer period.

### 3.6 Analysis of influencing factors of nilotinib plasma concentration

Univariate linear regression analysis was performed to evaluate the associations between nilotinib plasma concentrations and clinical variables. The results showed that only treatment duration was significantly associated with plasma concentration (B = 113.150, P = 0.036), while PLT approached but did not reach statistical significance (B = 1.294, P = 0.073). No significant associations were observed for age, sex, dosage, ALT, AST, TBIL, Cr, NEUT, or Hb (P > 0.05; Supplementary Table S4).

As univariate linear regression identifies only independent associations without adjusting for confounding factors, variables with P < 0.05 (treatment duration, P = 0.036), those approaching the significance threshold (PLT, P = 0.073), and metabolically relevant parameters (TBIL and Hb) were included in the multivariate linear regression model to assess correlations with plasma concentrations. The results showed that although the overall model did not reach statistical significance (F = 1.926, P = 0.111), treatment duration remained a significant independent predictor of nilotinib concentrations (B = 112.777, P = 0.029). No significant associations were observed for TBIL, Hb, or PLT (P > 0.05). These findings suggest that, apart from treatment duration, the included variables were insufficient to establish a robust predictive model.

## 4 Discussion

Nilotinib, a second-generation BCR::ABL1 inhibitor, significantly improves clinical outcomes in imatinib-resistant or intolerant CML patients and is recommended as first-line therapy for intermediate-to high-risk CML by the NCCN (2024) (Shah et al., 2024). However, interindividual variability in plasma protein binding, drug absorption, and CYP3A4-mediated metabolism (Alves et al., 2021; García-Gutiérrez and Hernández-Boluda, 2019) contributes to pharmacokinetic heterogeneity, leading to ongoing debates regarding the concentration-efficacy relationship. In this study, analysis of nilotinib plasma concentrations in 121 CML patients at Nanfang Hospital of Southern Medical University revealed substantial interindividual variability (25.00–2,509.46 ng mL^-1^), although the primary distribution range was consistent with internationally reported values (Larson et al., 2012; Verheijen et al., 2017; Wang et al., 2018). These findings support the need for further research into TDM.

Current evidence indicates a complex and controversial relationship between nilotinib plasma concentrations and clinical efficacy. A meta-analysis by Garcia-Ferrer et al. (2019) found no statistically significant difference in mean concentrations between patients who achieved MMR (1,075.2 ± 374.0 ng mL^-1^) and non-responders (1,025.7 ± 408.4 ng mL^-1^) (P = 0.536). Similarly, a study by Allegra et al. (2023) reported no significant correlation between nilotinib concentrations and 12-month MMR rates.

However, a critical methodological concern arises in exposure-response studies of TDM-managed cohorts: dose adjustments driven by therapeutic monitoring—specifically, escalation in patients with low exposure or reduction in those with high-exposure (Yates et al., 2020) —may artificially narrow the observed exposure range and consequently obscure the true pharmacokinetic-pharmacodynamic relationship. To address this potential bias, we applied dose normalization (C_norm_ = C_min_/(Dose/600 mg)), which estimates exposure under standardized dosing. Crucially, the efficacy association persisted after normalization: the identical optimal threshold (636.99 ng mL^-1^L) for MMR prediction was maintained, and C_norm_ remained significantly higher in responders (P < 0.05). This consistency suggests that while TDM practices attenuated statistical associations (AUC decreased by 5.5%), the 636.99 ng mL^-1^ threshold reflects a physiologically relevant efficacy target independent of dosing adjustments.

In contrast, our study demonstrated significantly higher concentrations in the effective groups compared to the ineffective groups (1,036.40 ± 463.67 vs. 737.14 ± 518.97 ng mL^-1^; P < 0.001), with MMR achieved at concentrations >636.99 ng mL^-1^. This threshold is closely aligned with findings from Fukuda et al. (Fukuda, 2022), who proposed an early-phase target of 619 ng mL^-1^. Notably, Fukuda’s cohort study focused on 3-month concentrations, while Garcia-Ferrer’s meta-analysis incorporated longitudinal data, suggesting a decreasing predictive value over time. The consistency between our findings and those of Fukuda highlights the importance of the pharmacokinetic window during the initial months of treatment. To enhance the predictive accuracy of therapeutic efficacy, comprehensive evaluations should also consider patient-specific genetic profiles (e.g., BCR::ABL1 mutation subtypes) and metabolic characteristics (e.g., CYP3A4 enzymatic activity) (Garcia-Ferrer et al., 2019; Miura, 2015; Tian et al., 2018).

According to the 2016 European LeukemiaNet (ELN) recommendations (Steegmann et al., 2016), most CML patients experience treatment-emergent ADRs during early-phase TKI therapy, predominantly of mild to moderate severity. In this study, the overall incidence of ADRs was 76.9%, with no significant association observed with plasma concentrations (P = 0.288). Grade 1–2 ADRs predominated (65.29%), while grade 3–4 events were infrequent (11.57%). However, this finding aligns with the established safety profile of nilotinib, characterized by frequent low-grade ADRs that are typically manageable. A Korean post-marketing surveillance study reported an overall ADR incidence of 61.3% for nilotinib (Ahn et al., 2022). Importantly, our data corroborate these observations, with 65.29% of patients experiencing only mild-to-moderate (Grade 1–2) events that did not necessitate dose modification or treatment discontinuation. The mean nilotinib concentration in patients with ADRs (934.90 ± 527.89 ng mL^-1^) was higher than that in patients without ADRs (796.39 ± 434.23 ng mL^-1^), consistent with the findings of the meta-analysis by Garcia-Ferrer et al. (2019). Compared with imatinib, nilotinib presents a higher risk of elevated TBIL (Carneiro et al., 2015). A comprehensive meta-analysis (Teo et al., 2013) reported a significantly increased risk of high-grade (grade≥3) hepatotoxicity in cancer patients receiving TKIs compared with controls. Notably, our study demonstrated a concentration-dependent risk of hyperbilirubinemia: the high-concentration group exhibited a significantly higher incidence of elevated TBIL (50.0%, P = 0.030), suggesting a potential alert threshold of 1,290.34 ng mL^-1^. Pharmacogenetic analyses have implicated uridine diphosphate glucuronosyltransferase 1A1 (UGT1A1) polymorphisms in nilotinib-induced hyperbilirubinemia (Singer et al., 2007), consistent with previous reports of TKI-associated hepatotoxicity (Teo et al., 2013). Although a meta-analysis indicated a higher incidence of rash in nilotinib-treated patients compared with those receiving imatinib (Li et al., 2023), no concentration-dependent relationship for rash was observed in the present study (13.2%, P = 0.682). In our cohort, the most common hematologic ADRs were anemia (25.7%) and thrombocytopenia (13.2%), with grade 3–4 incidences of 1.7% and 2.5%, respectively. Compared with other clinical trials (de Tute et al., 2024; He et al., 2023), our study reported a lower incidence of grade 3–4 hematologic toxicities, which may be attributable to the small sample size and potential racial differences. Despite comparable overall ADR rates across different concentration strata, clinicians remain vigilant for concentration-dependent toxicities in patients with high drug exposure and consider dosage optimization through TDM.

Nilotinib is primarily metabolised in the liver via CYP3A4-mediated oxidation and hydroxylation pathways (Tian et al., 2018). Univariate regression analysis revealed a significant positive correlation between treatment duration and plasma concentrations (B = 113.150, P = 0.036), suggesting that prolonged therapy may increase drug exposure through CYP3A4 saturation or cumulative effects. This pharmacokinetic finding is strongly supported by our clinical observation that the effective group had a significantly longer treatment duration compared to the ineffective group (P < 0.001). This disparity likely reflects the natural course of therapy: patients who achieve a molecular response are maintained on nilotinib, leading to longer cumulative exposure. Conversely, patients with primary resistance or treatment failure are often switched to alternative therapies, resulting in a shorter observed treatment duration. Thus, the longer treatment duration in the effective group may be both a cause and a consequence of successful therapy. Demographic variables such as sex (P = 0.534) and age (P = 0.733) showed no significant effect, consistent with previous studies reporting 10%–20% higher exposure in women than in men, a difference below the clinical threshold for dosage adjustment (Tian et al., 2018). Physiologically based pharmacokinetic (PBPK) modelling in paediatric studies (Heimbach et al., 2019; Hijiya et al., 2020), which incorporated the ontogeny of metabolic enzymes and physiological parameters, demonstrated comparable steady-state exposures between children receiving 230 mg m^-2^ twice daily (bid) and adults receiving 400 mg bid, with no observed age-related variability, consistent with our findings. Although food intake is known to increase nilotinib bioavailability (Boons et al., 2018), dietary variables were not assessed in this study, underscoring the need for multifactorial predictive models in future research.

Variables with statistical significance (P < 0.05; treatment duration), near-significance (PLT, P = 0.073), and metabolic relevance (TBIL, Hb) in univariate analyses were included in multivariate linear regression to evaluate associations with nilotinib plasma concentrations. Although the overall model lacked statistical significance (F = 1.926, P = 0.111), treatment duration remained an independent predictor of plasma concentrations (B = 112.777, P = 0.029), whereas TBIL, Hb, and PLT showed no significant associations (all P > 0.05). Notably, the longitudinal analysis by Garcia-Ferrer et al. (2019) showed a decline in the predictive utility of plasma concentrations with extended follow-up, highlighting limitations in our current variable selection. These findings emphasize the need for larger sample sizes and incorporation of unmeasured covariates to improve model robustness.

Our study is limited by its single-centre, retrospective design and modest sample size (n = 121), which restricts the power of subgroup analyses, particularly for rare, serious adverse events. Key covariates, including CYP3A4 genotypes, BCR::ABL1 mutation status, and other pharmacogenetic factors (e.g., UGT1A1), and drug-drug interactions, were not accounted for, potentially limiting generalizability of the findings. Although the lack of systematic genetic data is a common constraint in real-world pharmacokinetic studies, we cannot fully rule out its potential confounding effect on cohort distribution and response outcomes. Future prospective, multicentre studies are needed to address these limitations and refine predictive models. Systematic reviews by Garcia-Ferrer et al. (2019) (n = 654) and Wang et al. (2018) (n = 26) concluded that current evidence does not sufficiently support the routine use of therapeutic drug monitoring (TDM), a concern mirrored by our findings of limited threshold specificity. Nevertheless, the prospective data from Fukuda (2022) support the value of early-phase concentration monitoring and suggest that future studies should focus on dynamic concentration profiles during the initial treatment phase rather than relying on static thresholds. Our analysis acknowledges important constraints regarding prognostic factors. Due to incomplete documentation of baseline risk stratification (e.g., Sokal scores) in this retrospective cohort, we could not adjust for these established predictors in multivariate models. This limitation, common in real-world TDM studies (Yates et al., 2020), may influence the precision of exposure-response estimates but does not invalidate the identified threshold given its consistency with data (Fukuda, 2022; Garcia-Ferrer et al., 2019).

## 5 Conclusion

Our study concludes that TDM of nilotinib provides actionable guidance for optimising CML therapy, with particular emphasis on early phase concentration targets >636.99 ng·mL-1 to ensure efficacy, while requiring vigilant monitoring of bilirubin levels in patients >1,290.34 ng·mL-1. To address TDM-inherent adjustment bias, we implemented a dose-standardized exposure metric, which confirmed the robustness of these thresholds. Clinical treatment requires the incorporation of dynamic TDM to enhance therapeutic effectiveness and reduce toxicity risks, advancing CML management into precision medicine by personalizing patient treatment.

## Data Availability

The original contributions presented in the study are included in the article/Supplementary Material, further inquiries can be directed to the corresponding authors.
