# Correction: Correlation analysis between plasma concentration of nilotinib and clinical efficacy and safety in patients with chronic myeloid leukemia: a single–center retrospective cohort study

**DOI:** 10.3389/fphar.2025.1699696

**Published:** 2025-10-14

**Authors:** Ying Liu, Taoyan Lin, Boxin Zhao, Yilei Li, Ping Zheng

**Affiliations:** ^1^ Department Pharmacy, Institute Nanfang Hospital, Organization Southern Medical University, Guangzhou, China; ^2^ Department Clinical Pharmacy Center, Institute Nanfang Hospital, Organization Southern Medical University, Guangzhou, China

**Keywords:** nilotinib, chronic myeloid leukemia, plasma concentration, TDM, efficacy, safety

The **Equal contribution** of authors Ying Liu and Taoyan Lin was omitted in the published article. The statement has now been added as “These authors contributed equally to this work and share first authorship”.

Additionally, the funder “the GuangDong Pharmaceutical Association (Nos. 2023JZ04, 2024A02036, 2025HJB01006, 2025A02036) to YL” was erroneously omitted in the published article.

The original article has been updated.

