# Correction: Correlation analysis between plasma concentration of nilotinib and clinical efficacy and safety in patients with chronic myeloid leukemia: a single–center retrospective cohort study

**DOI:** 10.3389/fphar.2025.1730637

**Published:** 2025-11-14

**Authors:** Ying Liu, Taoyan Lin, Boxin Zhao, Yilei Li, Ping Zheng

**Affiliations:** 1 Department Pharmacy, Institute Nanfang Hospital, Organization Southern Medical University, Guangzhou, China; 2 Department Clinical Pharmacy Center, Institute Nanfang Hospital, Organization Southern Medical University, Guangzhou, China

**Keywords:** nilotinib, chronic myeloid leukemia, plasma concentration, TDM, efficacy, safety

The **funding statement** was erroneously written as “This study is supported by the GuangDong Pharmaceutical Association (Nos. 2023JZ04, 2024A02036, 2025HJB01006, 2025A02036) to YL.” This has now been corrected to the following: “This study is supported by the GuangDong Pharmaceutical Association (No. 2024A02036 and 2025HJB01006 to YTL; No. 2023JZ04 and 2025A02036 to PZ).”

The original article has been updated.

