# Supplementary material for: Correlation analysis between plasma concentration of nilotinib and clinical efficacy and safety in patients with chronic myeloid leukemia: a single–center retrospective cohort study
Source: Front Pharmacol. 2025 Sep 5;16:1676800. doi: 10.3389/fphar.2025.1676800 (PMC12446310; doi:10.3389/fphar.2025.1676800)
Supplement: Supplementary file 1 [file Table1.docx]

Supplementary Material

# Supplementary Tables

## Supplementary Table S1

| Table S1 Patient characteristics | | | |
| --- | --- | --- | --- |
| Variables | Effective Group (N=67) | Ineffective Group (N=54) | P-value |
| SEX, n (%) |  |  | 0.468 |
| Female | 22(40.7) | 23(34.3) |  |
| Male | 32(59.3) | 44(65.7) |  |
| Mean age | 37.48±11.58 | 36.76±14.66 | 0.843 |
| Prior treatment, n (%) |  |  | 0.312 |
| Imatinib | 11(36.7) | 19(63.3) |  |
| Comorbidity, n (%) |  |  | 0.433 |
| Hepatitis B | 7(63.6) | 4(0) |  |
| Hyperthyroidism | 2(100) | 0(0) |  |
| Thalassemia | 1(100) | 0(0) |  |
| COVID-19 | 1(100) | 0(0) |  |
| Dosage (mg) , n (%) |  |  | 0.273 |
| 150 | 0(0) | 2(3.7) |  |
| 300 | 0(0) | 4(7.4) |  |
| 400 | 0(0) | 1(1.9) |  |
| 450 | 5(7.5) | 0(0) |  |
| 600 | 59(88.1) | 45(83.3) |  |
| 750 | 0(0) | 1(1.9) |  |
| 800 | 3(4.5) | 1(1.9) |  |
| Treatment Duration (months), n (%) |  |  | 0.004 |
| 0~6 | 14(11.6) | 24(19.8) |  |
| 6~12 | 8(6.6) | 10(8.3) |  |
| >12 | 45(37.2) | 20(16.5) |  |

## Supplementary Table S2

Table S2 Overall Incidence and Severity Distribution of ADRs

| ADRs | Grade 1-2 n(%) | Grade 3-4 n(%) | Overall n(%) |
| --- | --- | --- | --- |
| Rash | 13(10.7) | 3(2.5) | 16(13.2) |
| Headache | 0(0) | 1(0.8) | 1(0.8) |
| Myalgia | 0(0) | 2(1.7) | 2(1.7) |
| ALT Increased | 31(25.6) | 1(0.8) | 32(26.4) |
| AST Increased | 21(17.4) | 0(0) | 21(17.4) |
| Hyperbilirubinemia | 33(27.3) | 1(0.8) | 34(28.1) |
| Lipase Increased | 3(2.5) | 0(0) | 3(2.5) |
| Amylase | 3(2.5) | 0(0) | 3(2.5) |
| Triglyceride Increased | 10(8.3) | 0(0) | 10(8.3) |
| Cholesterol High | 29(24.0) | 0(0) | 29(24.0) |
| Thrombocytopenia | 13(10.7) | 3(2.5) | 16(13.2) |
| Anemia | 29(24.0) | 2(1.7) | 31(25.7) |
| Neutropenia​ | 6(5.0) | 2(1.7) | 8(6.7) |

## Supplementary Table S3

Table S3 Incidence and Correlation Analysis of ADRs by Different Concentration Ranges

| Indicators | Low concentration  (N=40) | Medium concentration  (N=53) | High concentration  (N=28) | P-value |
| --- | --- | --- | --- | --- |
| ADR, n (%) |  |  |  | 0.792 |
| No ADRs | 11(27.5) | 11(20.8) | 6(21.4) |  |
| Grade 1-2 | 23(57.5) | 37(69.8) | 19(67.9) |  |
| Grade 3-4 | 6(15.0) | 5(9.4) | 3(10.7) |  |
| Rash, n (%) |  |  |  | 0.682 |
| No ADRs | 36(90.0) | 47(88.7) | 22(78.6) |  |
| Grade 1-2 | 3(7.5) | 5(9.4) | 5(17.9) |  |
| Grade 3-4 | 1(2.5) | 1(1.9) | 1(3.6) |  |
| Headache, n (%) |  |  |  | 0.524 |
| No ADRs | 40(100.0) | 52(98.1) | 28(100.0) |  |
| Grade 1-2 | 0(0) | 0(0) | 0(0) |  |
| Grade 3-4 | 0(0) | 1(1.9) | 0(0) |  |
| Myalgia, n (%) |  |  |  | 0.271 |
| No ADRs | 40(100.0) | 51(96.2) | 28(100.0) |  |
| Grade 1-2 | 0(0) | 0(0) | 0(0) |  |
| Grade 3-4 | 0(0) | 2(3.8) | 0(0) |  |
| ALT Increased, n (%) |  |  |  | 0.614 |
| No ADRs | 32(80.0) | 36(67.9) | 21(75.0) |  |
| Grade 1-2 | 8(20.0) | 16(30.2) | 7(25.0) |  |
| Grade 3-4 | 0(0) | 1(1.9) | 0(0) |  |
| AST Increased n (%) |  |  |  | 0.580 |
| No ADRs | 35(87.5) | 42(79.2) | 23(82.1) |  |
| Grade 1-2 | 5(12.5) | 11(20.8) | 5(4.9) |  |
| Grade 3-4 | 0(0) | 0(0) | 0(0) |  |
| Hyperbilirubinemia, n (%) |  |  |  | 0.030 |
| No ADRs | 32(80.0) | 41(77.4) | 14(50.0) |  |
| Grade 1-2 | 8(20.0) | 12(22.6) | 13(46.4) |  |
| Grade 3-4 | 0(0) | 0(0) | 1(3.6) |  |
| Lipase Increased, n (%) |  |  |  | 0.583 |
| No ADRs | 39(97.5) | 51(96.2) | 28(100.0) |  |
| Grade 1-2 | 1(2.5) | 2(3.8) | 0(0) |  |
| Grade 3-4 | 0(0) | 0(0) | 0(0) |  |
| Amylase Increased, n (%) |  |  |  | 0.583 |
| No ADRs | 39(97.5) | 51(96.2) | 28(100.0) |  |
| Grade 1-2 | 1(2.5) | 2(3.8) | 0(0) |  |
| Grade 3-4 | 0(0) | 0(0) | 0(0) |  |
| Triglyceride Increased, n (%) |  |  |  | 0.917 |
| No ADRs | 37(92.5) | 48(90.6) | 26(92.9) |  |
| Grade 1-2 | 3(7.5) | 5(9.4) | 2(7.1) |  |
| Grade 3-4 | 0(0) | 0(0) | 0(0) |  |
| Cholesterol High, n (%) |  |  |  | 0.809 |
| No ADRs | 31(77.5) | 41(77.4) | 20(71.4) |  |
| Grade 1-2 | 9(22.5) | 12(22.6) | 8(28.6) |  |
| Grade 3-4 | 0(0) | 0(0) | 0(0) |  |
| Thrombocytopenia, n (%) |  |  |  | 0.082 |
| No ADRs | 33(82.5) | 49(92.5) | 23(82.1) |  |
| Grade 1-2 | 4(10.0) | 4(7.5) | 5(17.9) |  |
| Grade 3-4 | 3(7.5) | 0(0) | 0(0) |  |
| Anemia, n (%) |  |  |  | 0.357 |
| No ADRs | 29(72.5) | 39(73.6) | 22(78.6) |  |
| Grade 1-2 | 9(22.5) | 14(26.4) | 6(21.4) |  |
| Grade 3-4 | 2(5.0) | 0(0) | 0(0) |  |
| Neutropenia​, n (%) |  |  |  | 0.155 |
| No ADRs | 37(92.5) | 51(96.2) | 25(89.3) |  |
| Grade 1-2 | 1(2.5) | 2(3.8) | 3(10.7) |  |
| Grade 3-4 | 2(5.0) | 0(0) | 0(0) |  |

## Supplementary Table S4

Table 4 Analysis of Influencing Factors of the Plasma Concentration of Nilotinib

| Variables | B | t | P-value |
| --- | --- | --- | --- |
| Age | 1.321 | 0.341 | 0.733 |
| Sex | 60.556 | 0.624 | 0.534 |
| Dosage | 0.292 | 0.525 | 0.600 |
| Treatment Duration (months) | 113.150 | 2.124 | 0.036 |
| ALT | -0.687 | -0.278 | 0.781 |
| AST | -0.365 | -0.085 | 0.932 |
| TBIL | -4.121 | -0.912 | 0.364 |
| CR | -1.827 | -1.154 | 0.251 |
| NEUT | -32.365 | -1.138 | 0.258 |
| Hb | -0.215 | -0.096 | 0.924 |
| PLT | 1.294 | 1.811 | 0.073 |

**
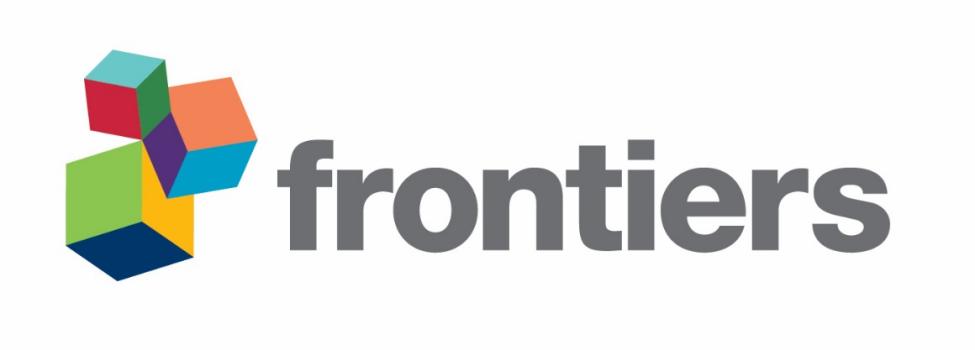
**
